# Supplementary material for: Multi‐dataset identification of innovative feature genes and molecular mechanisms in keratoconus
Source: J Cell Mol Med. 2024 Sep 19;28(18):e70079. doi: 10.1111/jcmm.70079 (PMC11412914; doi:10.1111/jcmm.70079)
Supplement: Supplementary file 2 — Material S2. [file JCMM-28-e70079-s002.zip › 7.GSEA analysis/6.SNORA74B/KEGG_WNT_SIGNALING_PATHWAY.html]

Details for gene set KEGG\_WNT\_SIGNALING\_PATHWAY[GSEA]

|  || Dataset | SNORA74B.group.cls#h\_versus\_l |
| Phenotype | group.cls#h\_versus\_l |
| Upregulated in class | l |
| GeneSet | KEGG\_WNT\_SIGNALING\_PATHWAY |
| Enrichment Score (ES) | -0.31822202 |
| Normalized Enrichment Score (NES) | -1.3518316 |
| Nominal p-value | 0.09090909 |
| FDR q-value | 0.32461137 |
| FWER p-Value | 0.93 |
Table: GSEA Results Summary

  

Fig 1: Enrichment plot: KEGG\_WNT\_SIGNALING\_PATHWAY      
 Profile of the Running ES Score & Positions of GeneSet Members on the Rank Ordered List

  

| SYMBOL | TITLE | RANK IN GENE LIST | RANK METRIC SCORE | RUNNING ES | CORE ENRICHMENT || 1 | SFRP1 | na | 2 | 1.422 | 0.0665 | No |
| 2 | CAMK2B | na | 141 | 0.835 | 0.1015 | No |
| 3 | NFATC2 | na | 1406 | 0.425 | 0.0849 | No |
| 4 | RAC3 | na | 1795 | 0.390 | 0.0919 | No |
| 5 | PPP3R2 | na | 2054 | 0.366 | 0.1016 | No |
| 6 | PRKCG | na | 2539 | 0.330 | 0.1030 | No |
| 7 | CAMK2A | na | 3838 | 0.265 | 0.0779 | No |
| 8 | CHP2 | na | 4857 | 0.230 | 0.0592 | No |
| 9 | DKK4 | na | 4878 | 0.229 | 0.0694 | No |
| 10 | WIF1 | na | 5513 | 0.208 | 0.0607 | No |
| 11 | DAAM2 | na | 5855 | 0.197 | 0.0601 | No |
| 12 | CER1 | na | 7307 | 0.153 | 0.0253 | No |
| 13 | WNT16 | na | 8300 | 0.129 | 0.0027 | No |
| 14 | CSNK1A1 | na | 8539 | 0.124 | 0.0016 | No |
| 15 | ROCK1 | na | 8851 | 0.117 | -0.0020 | No |
| 16 | WNT10B | na | 8944 | 0.115 | 0.0008 | No |
| 17 | PLCB3 | na | 9334 | 0.106 | -0.0055 | No |
| 18 | PLCB2 | na | 9573 | 0.101 | -0.0077 | No |
| 19 | CSNK2A1 | na | 9893 | 0.095 | -0.0125 | No |
| 20 | MAPK9 | na | 10427 | 0.085 | -0.0239 | No |
| 21 | WNT5A | na | 10441 | 0.084 | -0.0204 | No |
| 22 | CREBBP | na | 10525 | 0.083 | -0.0189 | No |
| 23 | LRP5 | na | 11242 | 0.071 | -0.0363 | No |
| 24 | PSEN1 | na | 11251 | 0.071 | -0.0332 | No |
| 25 | SFRP2 | na | 11258 | 0.071 | -0.0301 | No |
| 26 | CTBP1 | na | 11288 | 0.070 | -0.0276 | No |
| 27 | CAMK2G | na | 11900 | 0.062 | -0.0424 | No |
| 28 | PPP2R5A | na | 11922 | 0.061 | -0.0402 | No |
| 29 | PPP2R1B | na | 12000 | 0.060 | -0.0396 | No |
| 30 | WNT8A | na | 12290 | 0.056 | -0.0453 | No |
| 31 | WNT3 | na | 12537 | 0.053 | -0.0500 | No |
| 32 | VANGL1 | na | 12550 | 0.052 | -0.0479 | No |
| 33 | TCF7L2 | na | 12663 | 0.051 | -0.0487 | No |
| 34 | SMAD4 | na | 13214 | 0.045 | -0.0625 | No |
| 35 | SMAD2 | na | 13222 | 0.045 | -0.0607 | No |
| 36 | CTBP2 | na | 13433 | 0.042 | -0.0647 | No |
| 37 | CHD8 | na | 13572 | 0.041 | -0.0668 | No |
| 38 | FRAT2 | na | 13861 | 0.038 | -0.0734 | No |
| 39 | PRICKLE1 | na | 13949 | 0.036 | -0.0742 | No |
| 40 | RBX1 | na | 14044 | 0.035 | -0.0753 | No |
| 41 | WNT2 | na | 14134 | 0.034 | -0.0763 | No |
| 42 | CXXC4 | na | 14165 | 0.034 | -0.0755 | No |
| 43 | TBL1XR1 | na | 14209 | 0.034 | -0.0752 | No |
| 44 | FZD1 | na | 14343 | 0.032 | -0.0776 | No |
| 45 | LRP6 | na | 14448 | 0.031 | -0.0791 | No |
| 46 | MAPK10 | na | 14996 | 0.024 | -0.0938 | No |
| 47 | TBL1Y | na | 15195 | 0.023 | -0.0985 | No |
| 48 | CCND1 | na | 15223 | 0.022 | -0.0982 | No |
| 49 | SFRP4 | na | 15422 | 0.020 | -0.1030 | No |
| 50 | FZD5 | na | 15466 | 0.020 | -0.1033 | No |
| 51 | SFRP5 | na | 16158 | 0.013 | -0.1227 | No |
| 52 | SMAD3 | na | 16318 | 0.011 | -0.1267 | No |
| 53 | FZD6 | na | 16365 | 0.011 | -0.1276 | No |
| 54 | PPP2R5D | na | 16659 | 0.008 | -0.1357 | No |
| 55 | PORCN | na | 16660 | 0.008 | -0.1353 | No |
| 56 | PLCB4 | na | 16838 | 0.006 | -0.1401 | No |
| 57 | FBXW11 | na | 16931 | 0.005 | -0.1426 | No |
| 58 | CTNNBIP1 | na | 17449 | 0.000 | -0.1575 | No |
| 59 | ROCK2 | na | 17655 | -0.002 | -0.1633 | No |
| 60 | PPARD | na | 17698 | -0.003 | -0.1644 | No |
| 61 | CTNNB1 | na | 17783 | -0.003 | -0.1667 | No |
| 62 | TBL1X | na | 17852 | -0.004 | -0.1685 | No |
| 63 | GSK3B | na | 17871 | -0.004 | -0.1688 | No |
| 64 | FZD9 | na | 18101 | -0.007 | -0.1751 | No |
| 65 | WNT4 | na | 18195 | -0.008 | -0.1774 | No |
| 66 | SKP1 | na | 18228 | -0.009 | -0.1779 | No |
| 67 | RHOA | na | 18290 | -0.009 | -0.1792 | No |
| 68 | BTRC | na | 18505 | -0.012 | -0.1849 | No |
| 69 | WNT3A | na | 18601 | -0.013 | -0.1870 | No |
| 70 | FZD3 | na | 18707 | -0.014 | -0.1894 | No |
| 71 | RAC2 | na | 18892 | -0.016 | -0.1940 | No |
| 72 | NLK | na | 18915 | -0.016 | -0.1939 | No |
| 73 | PPP2R5C | na | 19299 | -0.021 | -0.2040 | No |
| 74 | PPP3R1 | na | 19504 | -0.023 | -0.2088 | No |
| 75 | TP53 | na | 19572 | -0.024 | -0.2096 | No |
| 76 | SIAH1 | na | 19796 | -0.026 | -0.2148 | No |
| 77 | RAC1 | na | 19881 | -0.027 | -0.2160 | No |
| 78 | NFATC3 | na | 20022 | -0.029 | -0.2187 | No |
| 79 | WNT9A | na | 20087 | -0.029 | -0.2192 | No |
| 80 | PPP2R5E | na | 20343 | -0.032 | -0.2251 | No |
| 81 | MAPK8 | na | 20414 | -0.033 | -0.2255 | No |
| 82 | CSNK2B | na | 20420 | -0.033 | -0.2241 | No |
| 83 | CUL1 | na | 20440 | -0.033 | -0.2231 | No |
| 84 | DAAM1 | na | 20566 | -0.035 | -0.2251 | No |
| 85 | AXIN1 | na | 20829 | -0.038 | -0.2309 | No |
| 86 | CSNK2A2 | na | 20870 | -0.039 | -0.2303 | No |
| 87 | DVL3 | na | 20946 | -0.039 | -0.2306 | No |
| 88 | CCND2 | na | 21016 | -0.040 | -0.2307 | No |
| 89 | PRKX | na | 21076 | -0.041 | -0.2305 | No |
| 90 | PPP2R1A | na | 21264 | -0.043 | -0.2339 | No |
| 91 | PPP2R5B | na | 21302 | -0.044 | -0.2329 | No |
| 92 | CHP1 | na | 21507 | -0.046 | -0.2367 | No |
| 93 | DVL1 | na | 21883 | -0.050 | -0.2452 | No |
| 94 | PPP3CB | na | 21942 | -0.051 | -0.2445 | No |
| 95 | PRKCA | na | 22239 | -0.055 | -0.2505 | No |
| 96 | CSNK1A1L | na | 22436 | -0.057 | -0.2535 | No |
| 97 | APC2 | na | 22646 | -0.060 | -0.2567 | No |
| 98 | PRKACA | na | 22659 | -0.060 | -0.2542 | No |
| 99 | SENP2 | na | 22990 | -0.065 | -0.2607 | No |
| 100 | PPP3CA | na | 23024 | -0.066 | -0.2586 | No |
| 101 | CACYBP | na | 23348 | -0.071 | -0.2646 | No |
| 102 | FZD4 | na | 23557 | -0.074 | -0.2672 | No |
| 103 | MAP3K7 | na | 23787 | -0.078 | -0.2702 | No |
| 104 | NFAT5 | na | 24769 | -0.094 | -0.2941 | No |
| 105 | FRAT1 | na | 24919 | -0.097 | -0.2939 | No |
| 106 | PPP2CA | na | 25151 | -0.101 | -0.2959 | No |
| 107 | CCND3 | na | 25274 | -0.103 | -0.2946 | No |
| 108 | WNT2B | na | 25314 | -0.104 | -0.2908 | No |
| 109 | JUN | na | 25317 | -0.104 | -0.2860 | No |
| 110 | EP300 | na | 25385 | -0.105 | -0.2831 | No |
| 111 | MYC | na | 25534 | -0.108 | -0.2823 | No |
| 112 | DKK1 | na | 25655 | -0.110 | -0.2806 | No |
| 113 | FZD8 | na | 25688 | -0.111 | -0.2764 | No |
| 114 | PPP2CB | na | 26235 | -0.122 | -0.2865 | No |
| 115 | NKD2 | na | 26387 | -0.125 | -0.2850 | No |
| 116 | DVL2 | na | 26618 | -0.130 | -0.2855 | No |
| 117 | WNT10A | na | 26979 | -0.139 | -0.2894 | No |
| 118 | TCF7L1 | na | 27185 | -0.144 | -0.2887 | No |
| 119 | RUVBL1 | na | 27333 | -0.148 | -0.2860 | No |
| 120 | FZD10 | na | 27856 | -0.161 | -0.2936 | No |
| 121 | NFATC4 | na | 27880 | -0.162 | -0.2867 | No |
| 122 | FOSL1 | na | 28417 | -0.177 | -0.2939 | No |
| 123 | VANGL2 | na | 28564 | -0.181 | -0.2896 | No |
| 124 | PPP3CC | na | 28713 | -0.186 | -0.2852 | No |
| 125 | TCF7 | na | 28810 | -0.189 | -0.2791 | No |
| 126 | WNT7B | na | 28850 | -0.190 | -0.2714 | No |
| 127 | WNT8B | na | 29836 | -0.219 | -0.2896 | No |
| 128 | APC | na | 30020 | -0.226 | -0.2843 | No |
| 129 | WNT9B | na | 30647 | -0.250 | -0.2907 | No |
| 130 | WNT7A | na | 31601 | -0.301 | -0.3042 | Yes |
| 131 | DKK2 | na | 31615 | -0.301 | -0.2904 | Yes |
| 132 | SOX17 | na | 32146 | -0.331 | -0.2903 | Yes |
| 133 | WNT1 | na | 32208 | -0.335 | -0.2764 | Yes |
| 134 | WNT6 | na | 32236 | -0.336 | -0.2615 | Yes |
| 135 | PRKACB | na | 32349 | -0.344 | -0.2486 | Yes |
| 136 | FZD2 | na | 32364 | -0.345 | -0.2329 | Yes |
| 137 | WNT5B | na | 32480 | -0.353 | -0.2197 | Yes |
| 138 | CAMK2D | na | 32655 | -0.364 | -0.2077 | Yes |
| 139 | PRKACG | na | 32698 | -0.368 | -0.1917 | Yes |
| 140 | PLCB1 | na | 32854 | -0.381 | -0.1784 | Yes |
| 141 | NFATC1 | na | 33261 | -0.420 | -0.1705 | Yes |
| 142 | FZD7 | na | 33265 | -0.421 | -0.1509 | Yes |
| 143 | PRKCB | na | 33882 | -0.508 | -0.1449 | Yes |
| 144 | NKD1 | na | 34026 | -0.538 | -0.1239 | Yes |
| 145 | AXIN2 | na | 34084 | -0.552 | -0.0997 | Yes |
| 146 | PRICKLE2 | na | 34301 | -0.613 | -0.0772 | Yes |
| 147 | MMP7 | na | 34307 | -0.615 | -0.0486 | Yes |
| 148 | LEF1 | na | 34351 | -0.633 | -0.0202 | Yes |
| 149 | WNT11 | na | 34430 | -0.665 | 0.0086 | Yes |
Table: GSEA details [plain text format]

  

Fig 2: KEGG\_WNT\_SIGNALING\_PATHWAY      
 Blue-Pink O' Gram in the Space of the Analyzed GeneSet

  

Fig 3: KEGG\_WNT\_SIGNALING\_PATHWAY: Random ES distribution      
 Gene set null distribution of ES for **KEGG\_WNT\_SIGNALING\_PATHWAY**
